# The Carbohydrate Environment During Differentiation Shapes the Functional Characteristics and Response to Acute Sugar Exposure of Human Intestinal Caco-2 Cells

**DOI:** 10.3390/nu18213555

**Published:** 2026-10-28

**Authors:** Mattia Di Nunzio, Elena Chiarello, Francesca Danesi, Francesco Capozzi, Alessandra Bordoni

**Affiliations:** 1 Department of Food, Environmental and Nutritional Sciences (DeFENS), University of Milan, Via Celoria 2, 20133 Milan, Italy; mattia.dinunzio@unimi.it; 2 Department of Agri-Food Sciences and Technologies (DISTAL), University of Bologna, Piazza Goidanich 60, 47521 Cesena, Italy; elena.chiarello28@gmail.com (E.C.); francesca.danesi@unibo.it (F.D.); francesco.capozzi@unibo.it (F.C.); 3 Interdepartmental Centre for Industrial Agri-Food Research (CIRI-AGRO), University of Bologna, Via Quinto Bucci 336, 47521 Cesena, Italy

**Keywords:** Caco-2 cells, carbohydrate environment, culture medium, cell differentiation, nutrient sensing, sugar transport, intestinal barrier

## Abstract

Background: The intestinal epithelium continuously adapts to changes in nutrient availability, yet whether the carbohydrate environment experienced during epithelial differentiation contributes to shaping the mature enterocyte functional characteristics remains largely unexplored. Objectives: This study investigated whether replacing glucose with fructose or sucrose during Caco-2 cell differentiation influences epithelial maturation, the expression of genes involved in intestinal sugar digestion and transport, and the cellular response to a subsequent acute sugar challenge. Methods: Caco-2 cells were differentiated for 23 days in medium containing glucose, fructose, or sucrose. Barrier function was monitored throughout differentiation by measuring transepithelial electrical resistance and paracellular permeability. At the end of differentiation, dome formation, expression of genes involved in sugar digestion and transport, and extracellular sugar distribution were evaluated. Differentiated cells were then exposed for 24 h to glucose, fructose, or sucrose to assess their adaptive response. Results: All carbohydrate conditions supported epithelial differentiation, although with distinct maturation kinetics. Sucrose promoted earlier barrier maturation, reduced dome formation, and generated a transcriptional profile that differed from those induced by glucose or fructose. The transcriptional response to acute sugar exposure depended on both the carbohydrate present during differentiation and that used for the subsequent challenge, whereas extracellular carbohydrate distribution remained largely unchanged among experimental groups. Conclusions: These findings demonstrate that the carbohydrate environment experienced during differentiation contributes to shaping the functional characteristics of intestinal epithelial cells. Beyond emphasizing the importance of carefully defining differentiation conditions in Caco-2-based studies, this work supports the concept that nutrient availability during epithelial maturation may represent an underappreciated determinant of intestinal epithelial adaptation.

## 1. Introduction

The intestinal epithelium is one of the most rapidly renewing tissues in the human body. Continuous self-renewal is sustained by intestinal stem cells residing at the base of the crypts, which continuously proliferate and differentiate into specialized epithelial cell lineages that migrate along the crypt–villus axis before being shed into the intestinal lumen. This tightly coordinated process maintains epithelial homeostasis by preserving barrier integrity while ensuring efficient nutrient digestion and absorption [1,2]. Far from being a static tissue, the intestinal epithelium exhibits remarkable structural and functional plasticity, allowing continuous adaptation to physiological and environmental cues [3]. Consequently, disturbances in epithelial renewal and differentiation have been implicated in the development of intestinal and systemic diseases, emphasizing the importance of identifying the factors that regulate enterocyte maturation and intestinal homeostasis [4,5].

Among the environmental factors influencing intestinal epithelial plasticity, the luminal nutritional environment plays a pivotal role. The intestinal epithelium is continuously exposed to dietary components that not only provide metabolic substrates but also regulate cellular signalling pathways involved in proliferation, differentiation, barrier function, and tissue homeostasis [6]. Accordingly, nutrients are increasingly recognized as bioactive molecules capable of modulating intestinal physiology through transcriptional and metabolic reprogramming rather than simply serving as energy sources [7].

This concept has emerged from both in vivo and in vitro studies showing that dietary components can influence epithelial renewal, barrier integrity, and intestinal adaptation under physiological and pathological conditions [8]. Fatty acids, amino acids, and carbohydrates have all been implicated in the regulation of signalling pathways controlling epithelial differentiation and metabolic homeostasis, suggesting that the nutritional environment experienced during enterocyte maturation may contribute to establishing the functional characteristics of the intestinal epithelium [9,10]. Nevertheless, the contribution of individual dietary nutrients to this process remains only partially understood.

Among dietary nutrients, carbohydrates are particularly interesting because they combine nutritional and signalling functions. Glucose, fructose, and sucrose are common dietary sugars that differ substantially in their digestion, absorption, and intracellular metabolism [11]. While glucose and fructose are directly transported across the intestinal epithelium by specific membrane transporters, sucrose must first be hydrolysed at the brush border by sucrase-isomaltase (SI) before its constituent monosaccharides become available for absorption [12].

Beyond these well-established differences in intestinal handling, dietary sugars actively regulate the expression of digestive enzymes and membrane transporters, thereby contributing to the adaptive regulation of carbohydrate absorption [13,14]. In addition, glucose and fructose activate nutrient-sensing pathways, including the carbohydrate-responsive element-binding protein (ChREBP), and have been implicated in the regulation of intestinal sugar transporter expression [15]. More recently, intestinal sweet taste receptors have also emerged as regulators of sugar sensing and transporter expression, supporting the concept that dietary sugars function not only as metabolic substrates but also as signalling molecules capable of modulating intestinal epithelial function [16].

Whether the carbohydrate environment experienced throughout epithelial differentiation contributes to programming, the functional characteristics of mature enterocytes has not yet been investigated. Dissecting the direct contribution of individual nutrients to intestinal epithelial programming is challenging in vivo because dietary interventions simultaneously affect microbiota, endocrine responses, immune signalling, and systemic metabolism. Consequently, well-characterized in vitro models remain indispensable for investigating the cell-autonomous effects of nutrients on epithelial differentiation.

Among these, the human intestinal Caco-2 cell line represents one of the most extensively validated models of the intestinal epithelium. Following confluence, Caco-2 cells spontaneously differentiate into polarized enterocyte-like monolayers that develop functional tight junctions, express brush-border enzymes, and acquire the major membrane transport systems involved in intestinal nutrient absorption [17]. Owing to these characteristics, differentiated Caco-2 cells are widely used to investigate epithelial barrier function, nutrient transport, and food–intestine interactions.

Importantly, accumulating evidence indicates that the functional characteristics acquired by Caco-2 cells is strongly influenced by the conditions under which differentiation occurs. Variations in serum source, amino acid supplementation, and other components of the differentiation medium have all been shown to modulate epithelial differentiation, barrier integrity, morphology, and transporter expression [9,18]. These observations suggest that differentiation conditions should not be regarded merely as technical aspects of cell culture, but as experimental variables capable of influencing the biological properties of the model itself.

Although the regulation of intestinal sugar transport by glucose and fructose has been extensively investigated, these studies have almost exclusively examined the short-term exposure of already differentiated intestinal epithelial cells to individual sugars [13,14,19]. Consequently, current knowledge mainly concerns the regulation of transporter expression and sugar absorption in mature enterocytes, whereas the potential contribution of the carbohydrate environment during the differentiation process itself has remained largely unexplored.

We hypothesized that the carbohydrate composition of the differentiation environment represents an underappreciated determinant of Caco-2 cell functional characteristics. Accordingly, this study was designed to determine whether replacing glucose with fructose or sucrose throughout Caco-2 differentiation influences epithelial barrier maturation, cell phenotype, and the expression of genes involved in intestinal sugar digestion and transport. Furthermore, we investigated whether the functional characteristics acquired during differentiation modulates the cellular response to a subsequent acute exposure to glucose, fructose, or sucrose. references.

## 2. Materials and Methods

### 2.1. Chemicals

Caco-2 cells were purchased from the American Type Culture Collection—ATCC (Manassas, VA, USA); Dulbecco’s Modified Eagle Medium (DMEM), penicillin, streptomycin, and Dulbecco’s phosphate-buffered saline (DPBS) were purchased from Lonza (Basel, Switzerland). Unless otherwise specified, all other chemicals and reagents were of analytical grade and purchased from Sigma-Aldrich (St. Louis, MO, USA).

### 2.2. Cell Culture Differentiation and Acute Sugar Exposure

Experiments were performed using Caco-2 cells (passages 50–60) seeded onto polyester Transwell^®^ inserts (0.4 μm pore size, 2 × 10^6^ pores/cm^2^, growth area 4.2 cm^2^; Corning Inc., Corning, NY, USA) at a density of 2 × 10^5^ cells/well. Cells were cultured in complete DMEM supplemented with 10% (*v*/*v*) fetal bovine serum, 1% (*v*/*v*) non-essential amino acids, 100 U/mL penicillin, and 100 μg/mL streptomycin. Depending on the experimental group, the medium contained 25 mM glucose, fructose, or sucrose. The concentration of 25 mM was selected based on the glucose concentration of high-glucose DMEM. Glucose was replaced by fructose or sucrose at the same molar concentration to investigate the effect of carbohydrate identity while maintaining an equimolar concentration of the sugar initially supplied. Phenol red (PR) (0.04 mM) was added only to the apical chamber to evaluate barrier permeability, whereas phenol red-free medium was used in the basolateral compartment.

Cells reached full confluence three days after seeding, which was defined as day 0 of differentiation. During the 23-day differentiation period, culture medium in both apical and basolateral chambers was replaced on days 0, 2, 6, 9, 13, 16, and 20. Twenty-four hours after each medium replacement (days 1, 3, 7, 10, 14, 17, and 21), barrier integrity and cell morphology were evaluated as described below.

On day 22, complete medium was replaced with serum-free DMEM containing the same sugar composition to avoid interference of serum components with the subsequent assays. After 24 h (day 23), barrier function, cell morphology, dome formation, cell viability, and cytotoxicity were evaluated. Culture media from both apical and basolateral chambers were collected and stored at −20 °C for subsequent analyses. Cells were either harvested for gene expression analysis or used for the acute sugar exposure experiment.

For the acute sugar exposure experiment, Caco-2 cells differentiated in glucose-, fructose- or sucrose-containing media (GLU-, FRU-, and SUC-cells, respectively) were incubated for an additional 24 h with serum-free complete DMEM containing either 25 mM glucose, fructose, or sucrose. After 24 h of exposure, barrier function, cell morphology, dome formation, cell viability, cytotoxicity, gene expression, and sugar concentrations in the culture media were evaluated.

**Figure 1:**
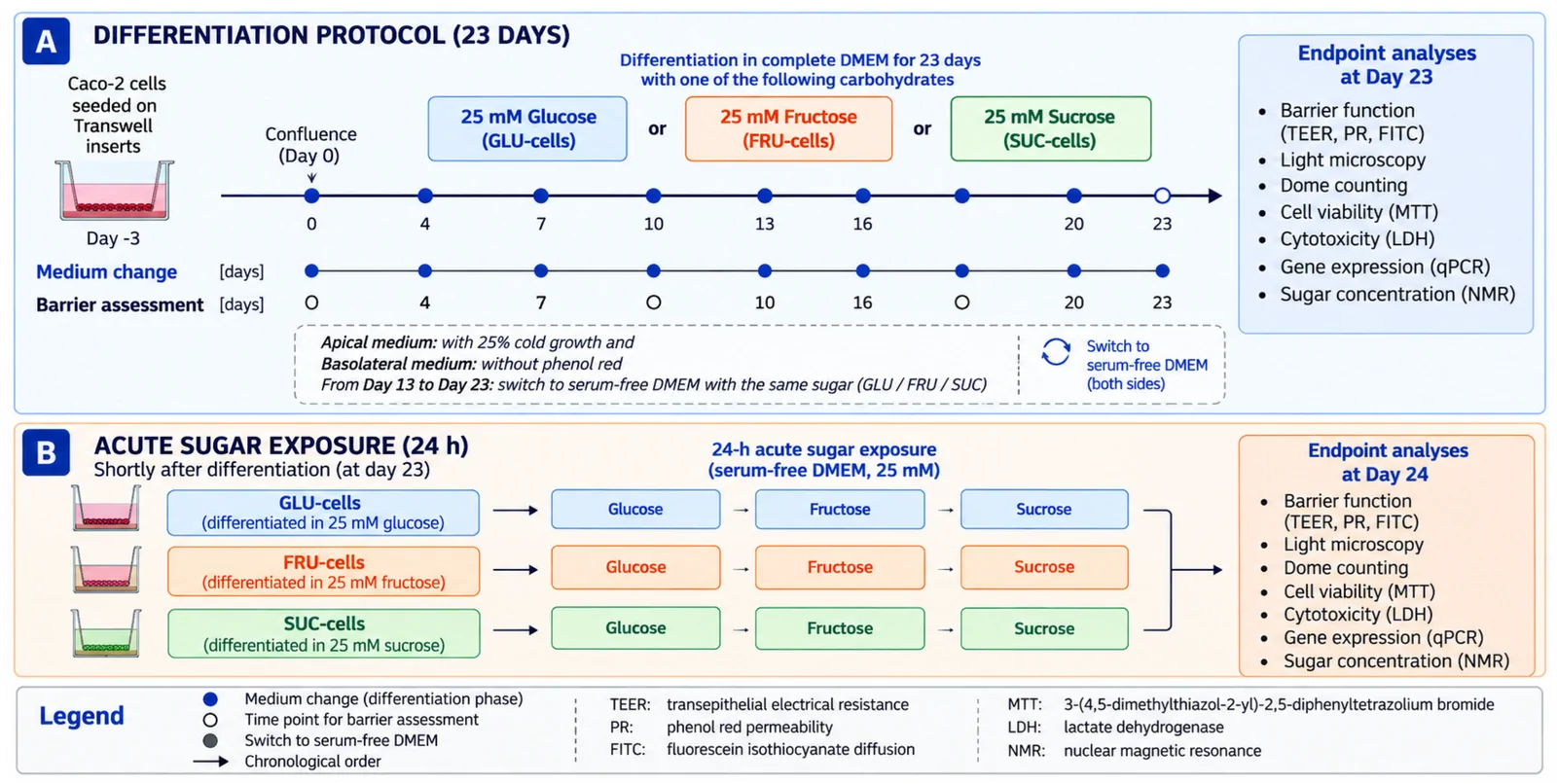
Schematic overview of experimental design. Panel (**A**): differentiation protocol; panel (**B**): acute sugar exposure.

A schematic overview of the experimental design is shown in Figure 1.

### 2.3. Assessment of Barrier Function and Cell Morphology

Barrier function was assessed by measuring transepithelial electrical resistance (TEER) and the paracellular diffusion of PR and fluorescein isothiocyanate (FITC). Cell morphology and monolayer integrity were evaluated by light microscopy.

#### 2.3.1. TEER Measurement

TEER was measured using a Millicell^®^ ERS voltohmmeter (Millipore, Burlington, MA, USA). TEER values between 1000 and 2000 Ω·cm^2^ were considered indicative of a fully differentiated monolayer [20].

#### 2.3.2. PR Permeability

Paracellular permeability was evaluated by measuring PR diffusion across the cell monolayer as previously described by Carullo et al. [21], with slight modifications. Briefly, 250 μL of medium were collected from the basolateral chamber and transferred to a 96-well plate. Absorbance was measured at 540 nm using an Infinite M200 microplate reader (Tecan, Männedorf, Switzerland), and PR concentration was calculated from an external calibration curve prepared in DMEM. PR permeability was expressed as percentage diffusion per hour. Values below 1%/h were considered acceptable for transport studies [22].

#### 2.3.3. FITC Permeability

FITC permeability was determined as previously described by De Marco et al. [23], with slight modifications. FITC solution (0.2 mg/mL, final concentration) was added to the apical chamber and, after 2 h, 250 μL were collected from the basolateral chamber. Fluorescence was measured (λ_ex_ = 485 nm; λ_em_ = 535 nm) using an Infinite F200 microplate reader (Tecan, Männedorf, Switzerland). FITC diffusion was quantified using an external calibration curve and expressed as percentage diffusion per hour.

#### 2.3.4. Light Microscopy

Cell morphology and monolayer integrity were examined throughout the experiments using an Eclipse Ti inverted microscope (Nikon Instruments, Florence, Italy) equipped with a 10× objective.

### 2.4. Cytotoxicity

Cell viability and cytotoxicity were evaluated by the methylthiazolyldiphenyltetrazolium bromide (MTT) assay and by measuring lactate dehydrogenase (LDH) activity in the apical culture medium.

#### 2.4.1. MTT Assay

Cell viability was determined by measuring the reduction of MTT to formazan, as previously described [24]. Absorbance was measured at 560 nm using an Infinite M200 microplate reader (Tecan, Männedorf, Switzerland). Cell viability was expressed as optical density.

#### 2.4.2. LDH Release

Cytotoxicity was assessed by measuring LDH activity released into the apical medium, following the oxidation of nicotinamide adenine dinucleotide (NADH) at 340 nm for 1 min, as previously described [25]. LDH activity was calculated using the extinction coefficient of NADH and expressed as mU/mL.

### 2.5. Domes Formation

Dome formation was evaluated by light microscopy using an Eclipse Ti inverted microscope (Nikon Instruments, Florence, Italy) equipped with a 10× objective [26]. Dome density was expressed as the number of domes per well.

### 2.6. Isolation of RNA and Quantitative Real-Time PCR (qPCR)

Cells were washed twice with 1 mL of warm DPBS, scraped into 1 mL of ice-cold DPBS, and collected by centrifugation (250× *g*, 5 min, 4 °C). Cell pellets were immediately processed for RNA extraction using the Direct-zol™ RNA MiniPrep kit (Zymo Research, Irvine, CA, USA), according to the manufacturer’s instructions. Three independent biological experiments were performed, each including two biological replicates. Replicates from the same experiment were pooled in pairs before RNA extraction. Total RNA was diluted to 40 ng/μL as previously described [27]. RNA concentration and purity were assessed using a NanoDrop ND-2000 spectrophotometer (Thermo Fisher Scientific, Wilmington, DE, USA), and samples were stored at −80 °C until analysis.

Reverse-transcription was performed using High-Capacity RNA-to-cDNA™ Kit (Applied Biosystems, Foster City, CA, USA), following manufacturer’s instruction. cDNA was subjected to qPCR using TaqMan™ Fast Advanced Master Mix (Applied Biosystems) with TaqMan™ Gene Expression Assay probes (Applied Biosystems) on a CFX Connect™ Real-Time PCR Detection System (Bio-Rad Laboratories; Hercules, CA, USA). TaqMan probes were as follows: sucrase-isomaltase (*SI*; Hs00356112_m1), solute carrier family 2 member 1 (*SLC2A1*, encoding GLUT1; Hs00197884_m1), solute carrier family 2 member 2 (*SLC2A2*, encoding GLUT2; Hs00165775_m1), solute carrier family 2 member 5 (*SLC2A5*, encoding GLUT5; Hs01086390_m1), and solute carrier family 5 member 1 (*SLC5A1*, encoding SGLT1; Hs01573790_m1).

Samples were run in duplicate using the following program: uracil-N glycosylase incubation at 50 °C for 2 min and polymerase activation at 95 °C for 20 s, followed by 40 cycles (95 °C for 3 s and 60 °C for 30 s). Data were analysed using CFX Maestro software (version 5.3; Bio-Rad Laboratories). Relative mRNA levels were calculated using the 2-ΔΔCq method, using glyceraldehyde-3-phosphate dehydrogenase (*GAPDH*; Hs99999905_m1), peptidylprolyl isomerase A (*PPIA*; Hs99999904_m1), and ribosomal protein lateral stalk subunit P0 (*RPLP0*; Hs99999902_m1) as reference genes. The stability of *GAPDH*, *PPIA*, and *RPLP0* across experimental conditions was evaluated using the Reference Gene Selection Tool of CFX Maestro software (version 5.3; Bio-Rad Laboratories), which applies the geNorm algorithm [28]. All three genes met the accepted stability criteria (M values < 0.5) and were therefore used for normalization.

### 2.7. ^1^H NMR Spectroscopy

Culture media collected from the apical and basolateral chambers were analysed by ^1^H NMR spectroscopy. Briefly, 500 μL of sample were mixed with 10 μL of a D_2_O solution containing 100 mM 2,2-dimethyl-2-silapentane-d6-5-sulfonate (DSS), resulting in a final DSS concentration of 9.09 mM. Spectra were acquired at 298 K on a Bruker US+ Avance III spectrometer operating at 600 MHz, equipped with a BBI-z probe and a B-ACS 60 sampler for automation (Bruker BioSpin, Karlsruhe, Germany). The residual HOD signal was suppressed using the noesygppr1d pulse sequence available in the Bruker pulse program library. Each spectrum was acquired using 32 K data points over a spectral width of 7183.908 Hz (12 ppm), with 256 transients. A recycled delay of 5 s, an acquisition time of 2.28 s, and a 90° pulse of 11.190 μs were used. The sum of the acquisition time and recycle delay was set to be more than five times longer than the longest longitudinal relaxation time measured for the selected carbohydrate resonances, which did not exceed 1.45 s.

The data were Fourier transformed and phase and baseline corrections were automatically performed using TopSpin version 4.5.0 (Bruker BioSpin, Karlsruhe, Germany). Signals were identified by comparing their chemical shifts and multiplicities with the Chenomx Profiler database (version 8.1, Edmonton, AB, Canada), published data, and spectra acquired from authentic standards of glucose, fructose, and sucrose under the same experimental conditions. Carbohydrate concentrations were determined by direct quantitative 1H NMR through integration of selected non-overlapping resonances assigned to each carbohydrate. Anomeric proton signals were used for glucose and sucrose, whereas well-resolved fructose resonances were selected for fructose quantification. Absolute concentrations were calculated from the ratio between the integrated area of each selected carbohydrate signal and that of the internal standard DSS, taking into account the number of contributing protons. The absence of relevant signal overlap for the selected resonances, as well as the reproducibility of the integrations, was verified on replicate spectra. Under the quantitative acquisition conditions used, this approach allowed direct concentration determination without analyte-specific calibration curves.

### 2.8. Statistical Analysis

Data are presented as means ± standard deviation (SD) of four independently treated Transwell inserts obtained from two independent cell culture experiments (two inserts per condition in each experiment). Data normality was assessed using the Shapiro–Wilk test, and homogeneity of variances was evaluated using the Brown–Forsythe test. Statistical analyses were performed using the one-way analysis of variance (ANOVA) followed by Tukey’s post hoc test, or by unpaired Student’s *t*-test, as appropriate. Time-course data for TEER, PR permeability, and FITC permeability were analysed by two-way analysis of variance (ANOVA), followed by the Tukey HSD test (*p* < 0.05), considering carbohydrate and time as variables. Differences were considered statistically significant at *p* < 0.05.

## 3. Results

### 3.1. Cell Differentiation in the Different Media

**Figure 2:**
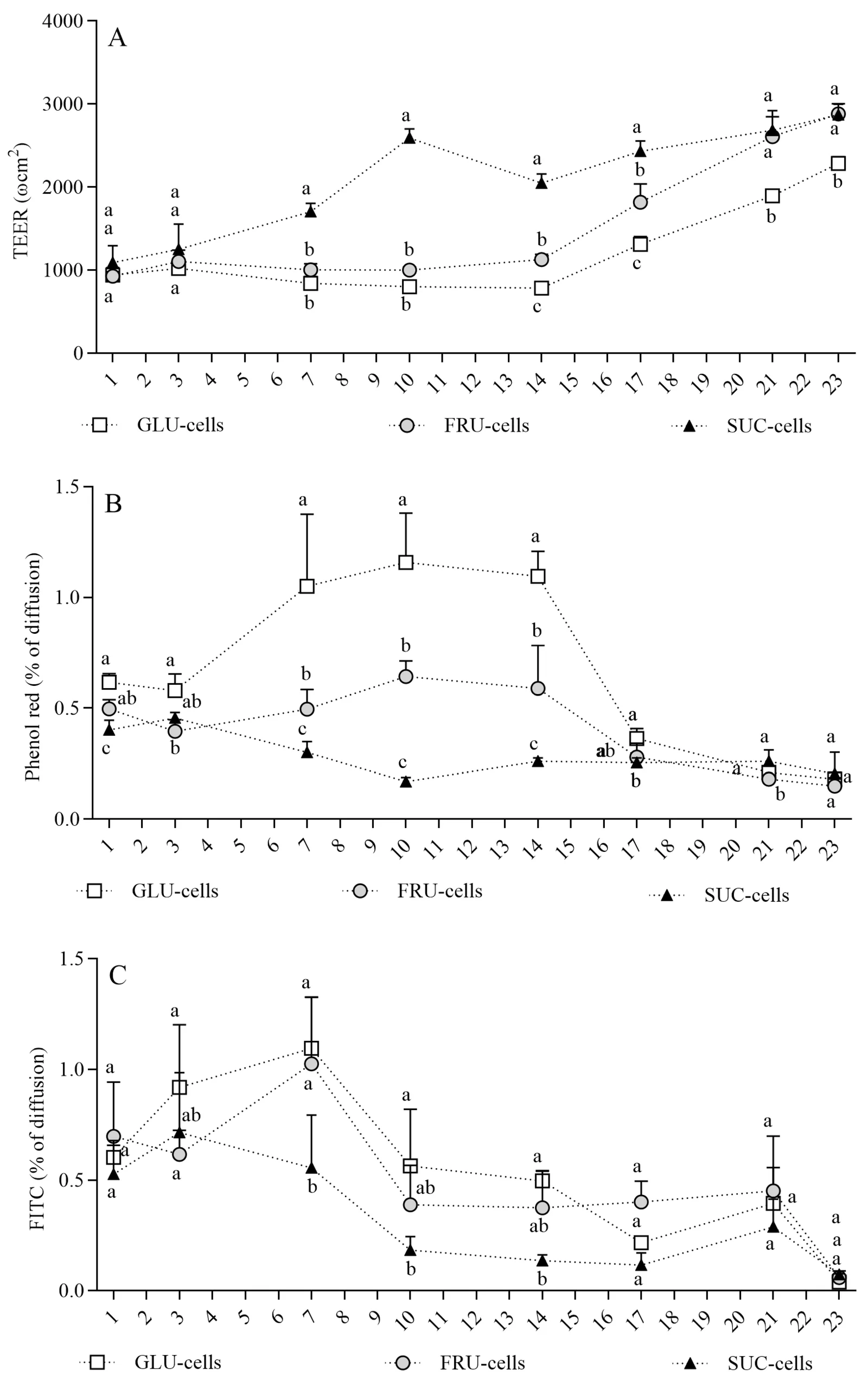
Time-course of barrier function during differentiation of GLU-, FRU-, and SUC-cells. Exposure. (**A**) Transepithelial electrical resistance (TEER), (**B**) phenol red (PR) permeability, and (**C**) fluorescein isothiocyanate (FITC) permeability measured throughout the 23-day differentiation period. GLU-, FRU-, and SUC-cells are indicated by white squares, grey circles, and black triangles, respectively. Data are expressed as Ω·cm^2^ (**A**) or percentage diffusion per hour (**B**,**C**) and are presented as means ± SD of four independently treated Transwell inserts obtained from two independent cell culture experiments (two inserts per condition in each experiment). Statistical analysis was performed by two-way ANOVA ((**A**,**B**) carbohydrate *p* < 0.001, time *p* < 0.001, carbohydrates*time *p* < 0.001; (**C**) carbohydrate *p* < 0.001, time *p* < 0.001, carbohydrates*time n.s.) followed by Tukey’ HSD post hoc test. Different letters indicate statistically significant differences among groups.

To investigate whether replacing glucose with fructose or sucrose affected Caco-2 cell differentiation, barrier function was monitored throughout the 23-day differentiation period by measuring TEER and the paracellular diffusion of PR and FITC (Figure 2).

Caco-2 cells differentiated successfully under all experimental conditions. At day 23, TEER values were markedly higher than those measured at day 1 in GLU-, FRU-, and SUC-cells. However, the sugar present in the differentiation medium significantly affected the kinetics of barrier maturation. Compared with GLU-cells, TEER increased earlier and reached significantly higher values in SUC-cells from day 7 onwards and in FRU-cells from day 14 onwards (Figure 2A).

Similarly, PR permeability progressively decreased during differentiation in all experimental groups. During the differentiation process, SUC-cells consistently showed lower PR permeability than GLU- and FRU-cells, whereas the latter displayed a transient increase between days 7 and 14. By day 21, however, PR permeability was comparable among the three groups (Figure 2B).

A similar trend was observed for FITC permeability. Although FITC diffusion decreased over time in all experimental conditions, SUC-cells exhibited significantly lower permeability than GLU- and FRU-cells at day 7 and remained significantly lower than GLU-treated cells at both days 10 and 14. No differences among groups were detected from day 17 (Figure 2C). Thus, although PR and FITC permeability converged at the end of differentiation, TEER remained significantly lower in GLU-cells than in FRU- and SUC-cells, indicating that the carbohydrate environment affected both the kinetics of barrier maturation and, to some extent, the final barrier characteristics.

Light microscopy confirmed the progressive formation of an intact cell monolayer throughout differentiation, with no evident morphological alterations or signs of cell damage in any experimental group.

### 3.2. Phenotypic and Molecular Characterization of Differentiated GLU-, FRU-, and SUC-Cells at the End of Differentiation

**Figure 3:**
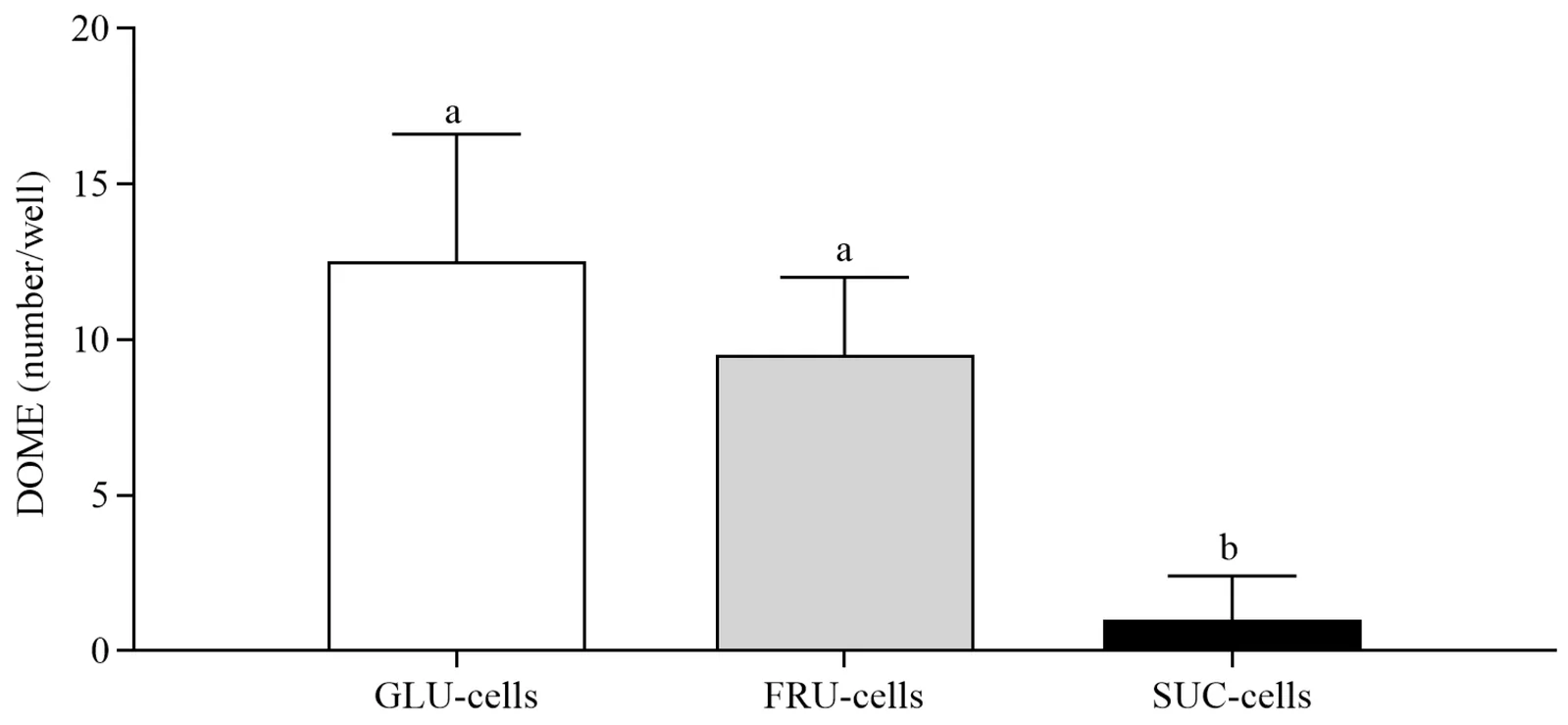
Dome formation in differentiated GLU-, FRU-, and SUC-cells. Dome density was evaluated at the end of the differentiation period (day 23) and is expressed as the number of domes per well. Data are presented as means ± SD of four independently treated Transwell inserts obtained from two independent cell culture experiments (two inserts per condition in each experiment). Statistical analysis was performed by one-way ANOVA (*p* < 0.001) followed by Tukey’s post hoc test. Different letters indicate statistically significant differences among groups.

Cell viability, assessed by the MTT assay and LDH release, did not differ among GLU-, FRU-, and SUC-cells. In contrast, dome density was significantly lower in SUC-cells than in the other experimental groups (Figure 3).

**Figure 4:**
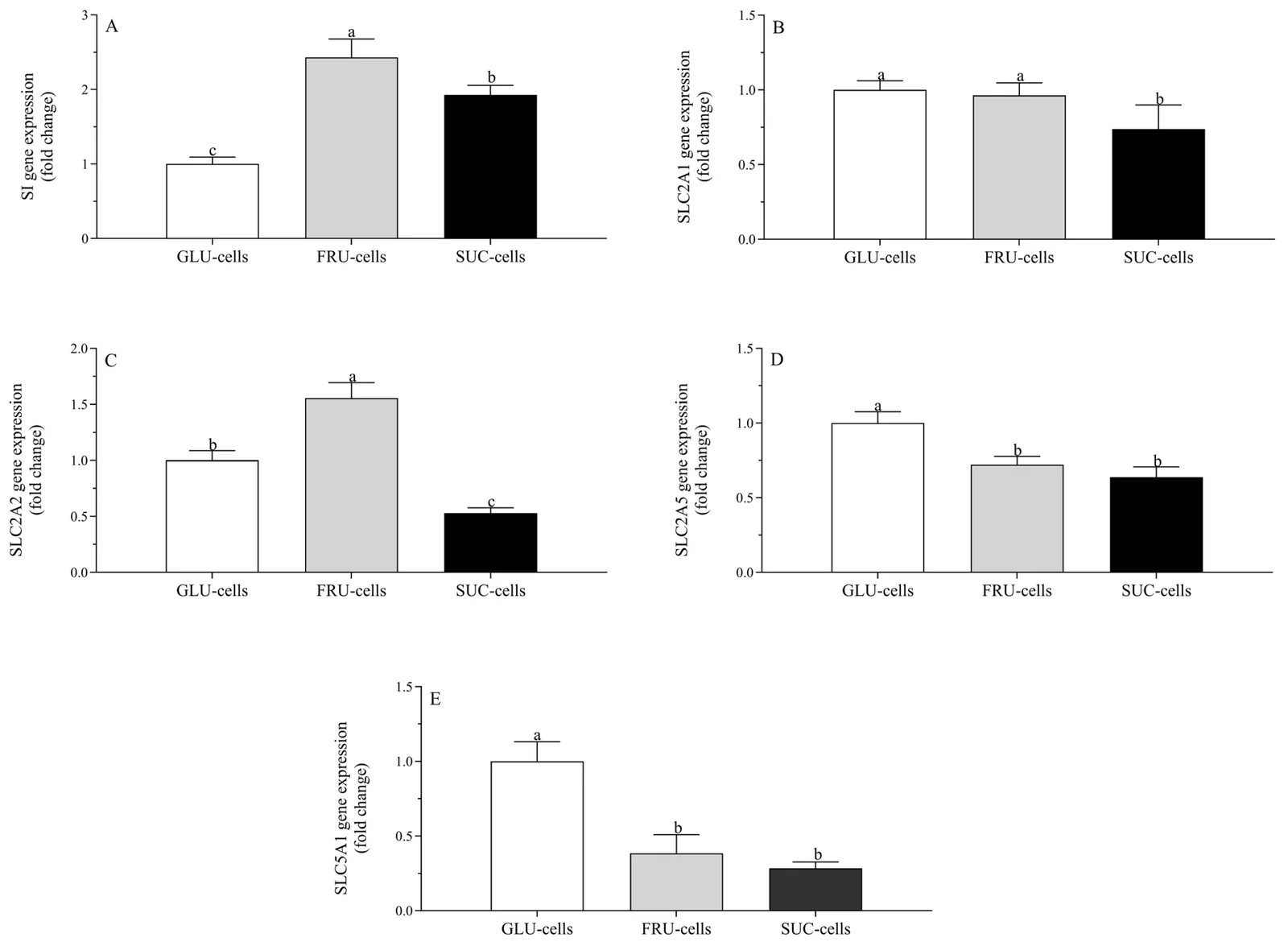
Expression of genes encoding sugar transporters and sucrase-isomaltase in differentiated GLU-, FRU-, and SUC-cells. Relative expression of (**A**) *SI*, (**B**) *SLC2A1*, (**C**) *SLC2A2*, (**D**) *SLC2A5*, and (**E**) *SLC5A1* at the end of the differentiation period (day 23). Gene expression is reported as fold change relative to GLU-cells (set to 1) and presented as means ± SD of three pooled samples per condition, each comprising two samples from one of three independent experiments (*n* = 3 per condition). Statistical analysis was performed by one-way ANOVA ((**A**,**C**,**D**) *p* < 0.001; (**B**) *p* < 0.01) followed by Tukey’s post hoc test. Different letters indicate statistically significant differences among groups.

The expression of *SI*, *SLC2A1*, *SLC2A2*, *SLC2A5*, and *SLC5A1* genes was markedly affected by the sugar present during differentiation (Figure 4). Compared with FRU- and SUC-cells, GLU-cells exhibited lower *SI* expression and higher expression of *SLC2A5* and *SLC5A1* (Figure 4A,D,E). *SLC2A2* expression was highest in FRU-cells, intermediate in GLU-cells, and lowest in SUC-cells (Figure 4C), whereas *SLC2A1* expression was comparable in GLU- and FRU-cells and significantly lower in SUC-cells (Figure 4B).

**Figure 5:**
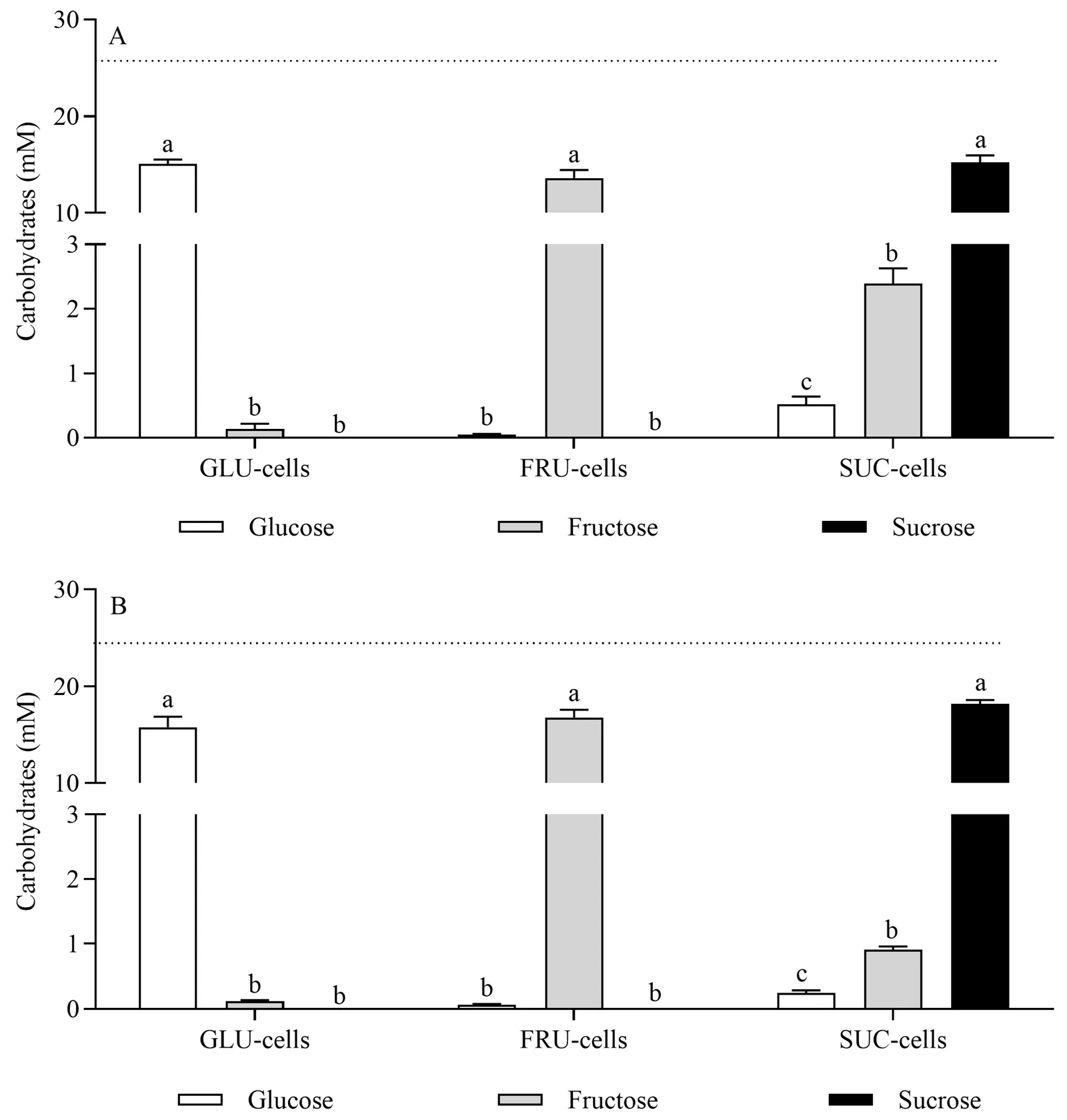
Sugar distribution in the apical and basolateral compartments of differentiated GLU-, FRU-, and SUC-cells. Glucose, fructose, and sucrose concentrations measured 24 h after the last medium replacement in the (**A**) apical and (**B**) basolateral compartments. Data are expressed as mM and presented as means ± SD of four independently treated Transwell inserts obtained from two independent cell culture experiments (two inserts per condition in each experiment). The dashed line indicates the initial sugar concentration in the culture medium. Statistical analysis was performed by one-way ANOVA (all *p* < 0.001) followed by Tukey’s post hoc test. Different letters indicate statistically significant differences among sugars within each differentiated cell type.

To evaluate whether these transcriptional differences were associated with changes in the extracellular distribution of carbohydrates, glucose, fructose, and sucrose concentrations were measured in the apical and basolateral compartments 24 h after the last medium replacement (Figure 5). In GLU- and FRU-cells, the supplemented monosaccharide was detected in both compartments, together with negligible amounts of the alternative monosaccharide. In SUC-cells, sucrose was detected in both chambers, whereas appreciable amounts of glucose and fructose were also present.

### 3.3. Adaptive Response of Differentiated GLU-, FRU-, and SUC-Cells to Acute Sugar Exposure

To determine whether the phenotypic differences acquired during differentiation influenced the cellular response to an acute sugar challenge, differentiated GLU-, FRU-, and SUC-cells were exposed for 24 h to glucose, fructose, or sucrose. Barrier function, cell phenotype, gene expression, and sugar distribution were then re-evaluated.

Twenty-four-hour exposure to glucose, fructose, or sucrose did not affect barrier function or cell morphology in any of the differentiated cell types. Likewise, cell viability and LDH release remained unchanged regardless of the sugar used for acute exposure.

**Figure 6:**
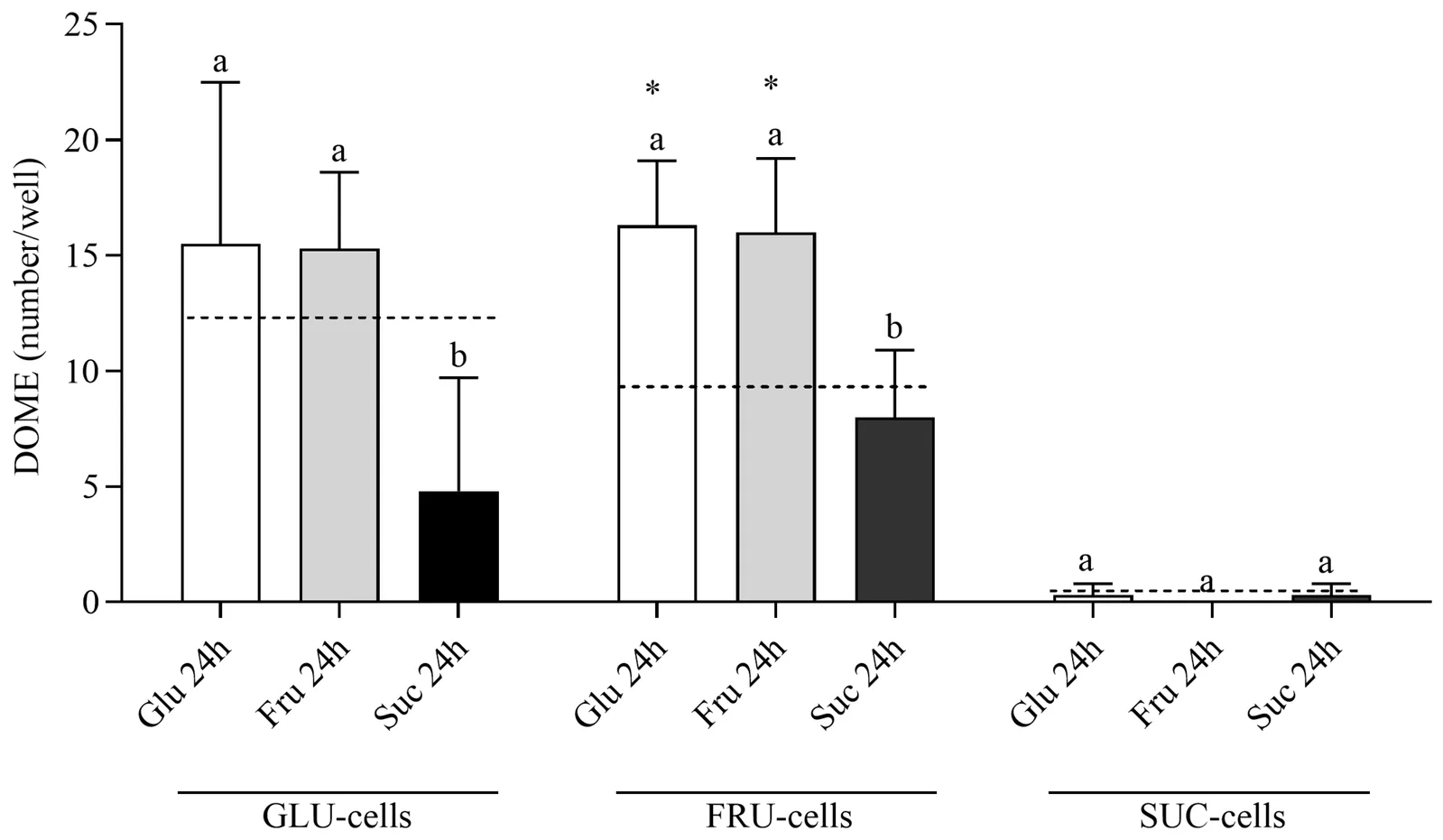
Dome formation after acute sugar exposure in differentiated GLU-, FRU-, and SUC-cells. Data are expressed as number of domes/well are means ± SD of four independently treated Transwell inserts obtained from two independent cell culture experiments (two inserts per condition in each experiment). The dashed line indicates dome formation in differentiated GLU-, FRU-, and SUC-cells. Statistical analysis was by the one-way ANOVA (GLU-cells: *p* < 0.05; FRU-cells *p* <0.01) followed by Tukey’s post hoc to compare the number of domes in each cell type. Different letters indicate statistically significant differences among sugar treatments within the same differentiated cell type. * indicate significant differences compared with the corresponding differentiated cells at day 23 (unpaired Student’s *t*-test).

In contrast, acute sugar exposure affected dome formation in a differentiation-dependent manner (Figure 6). In both GLU- and FRU-cells, sucrose exposure significantly reduced dome density compared with day 23. Conversely, exposure to glucose or fructose increased dome density in FRU-cells. In SUC-cells, dome density remained consistently low regardless of the sugar used for supplementation.

**Figure 7:**
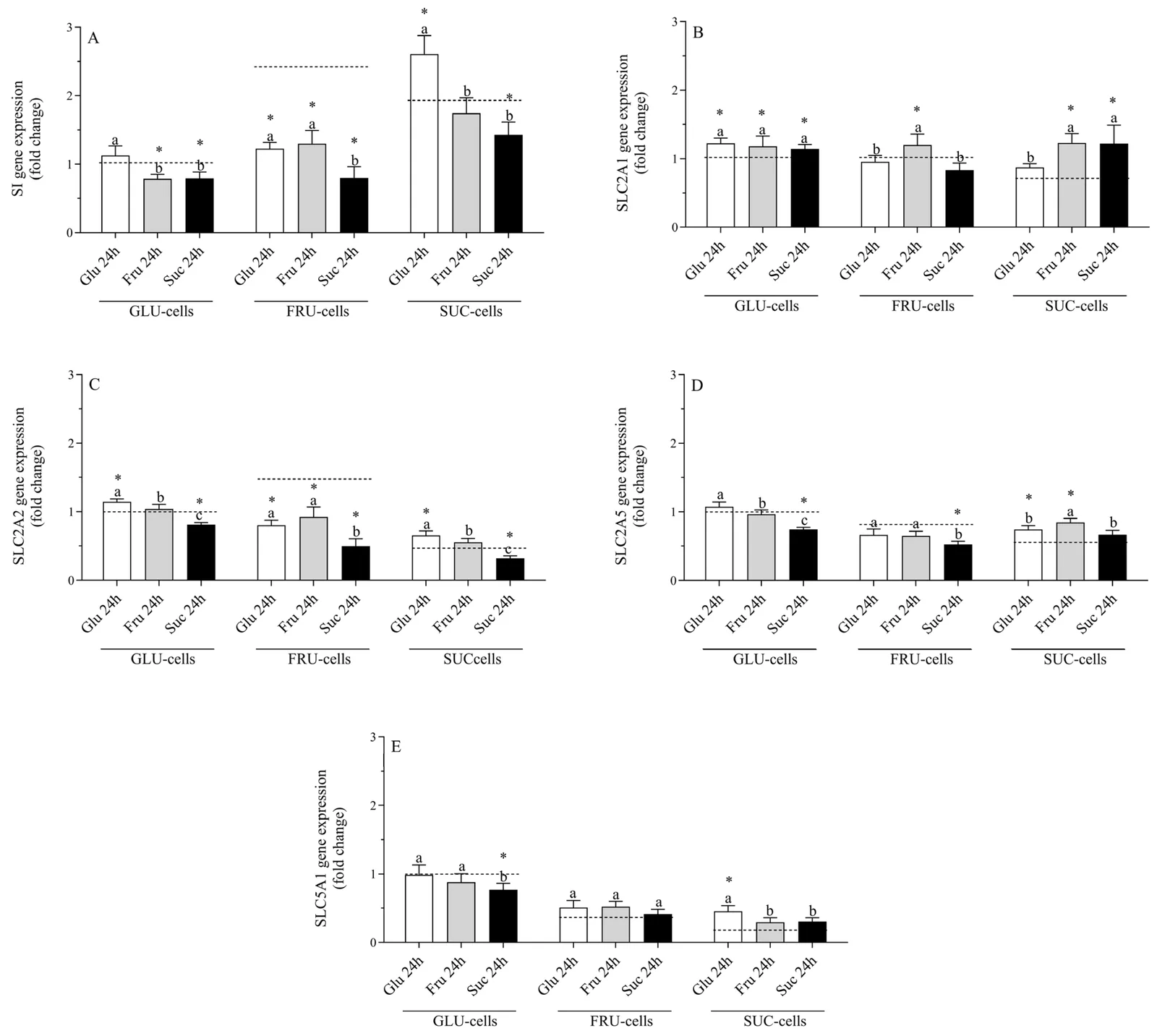
Effect of acute sugar exposure on expression of genes encoding sugar transporters and sucrase-isomaltase in differentiated GLU-, FRU-, and SUC-cells. Relative expression of (**A**) *SI*, (**B**) *SLC2A1*, (**C**) *SLC2A2*, (**D**) *SLC2A5*, and (**E**) *SLC5A1* after 24 h exposure to glucose, fructose, or sucrose. Gene expression is reported as fold change relative to the corresponding differentiated cells at day 23 (dashed line), which were used as the reference condition. Data are presented as means ± SD of three pooled samples per condition, each comprising two samples from one of three independent experiments (*n* = 3 per condition). Statistical analysis was performed by one-way ANOVA ((**A**,**C**) all *p* < 0.001; (**B**) FRU- and SUC-cells *p* < 0.01; (**D**) GLU- and SUC-cells *p* < 0.001, FRU-cells *p* < 0.01; (**E**) GLU-cells *p* < 0.05, SUC-cells *p* < 0.01) followed by Tukey’s post hoc test to compare the effects of the three sugars within each differentiated cell type. Different letters indicate statistically significant differences among sugar treatments within the same differentiated cell type. * indicate significant differences compared with the corresponding differentiated cells at day 23 (unpaired Student’s *t*-test).

The transcriptional response to acute sugar exposure depended on both the sugar present during differentiation and the sugar used for the subsequent acute exposure (Figure 7). Gene expression levels were compared with those measured in the corresponding cell type at the end of differentiation (day 23), which served as the reference condition.

SI expression generally decreased after fructose or sucrose exposure, irrespective of the differentiation medium, whereas glucose exposure produced little or no effect in GLU-cells and increased *SI* expression in both FRU- and SUC-cells (Figure 7A). *SLC2A1* and *SLC2A2* exhibited distinct responses to sugar exposure. *SLC2A1* expression was generally induced, particularly after fructose exposure, whereas *SLC2A2* expression was consistently reduced by sucrose and by all sugar treatments in FRU-cells (Figure 7B,C). Changes in *SLC2A5* and *SLC5A1* expression were more selective. Acute sucrose exposure reduced SLC2A5 expression in GLU- and FRU-cells, whereas glucose and fructose increased its expression in SUC-cells. *SLC5A1* expression was largely unaffected, with the exception of a decrease after sucrose exposure in GLU-cells and an increase after glucose exposure in SUC-cells (Figure 7D,E).

**Figure 8:**
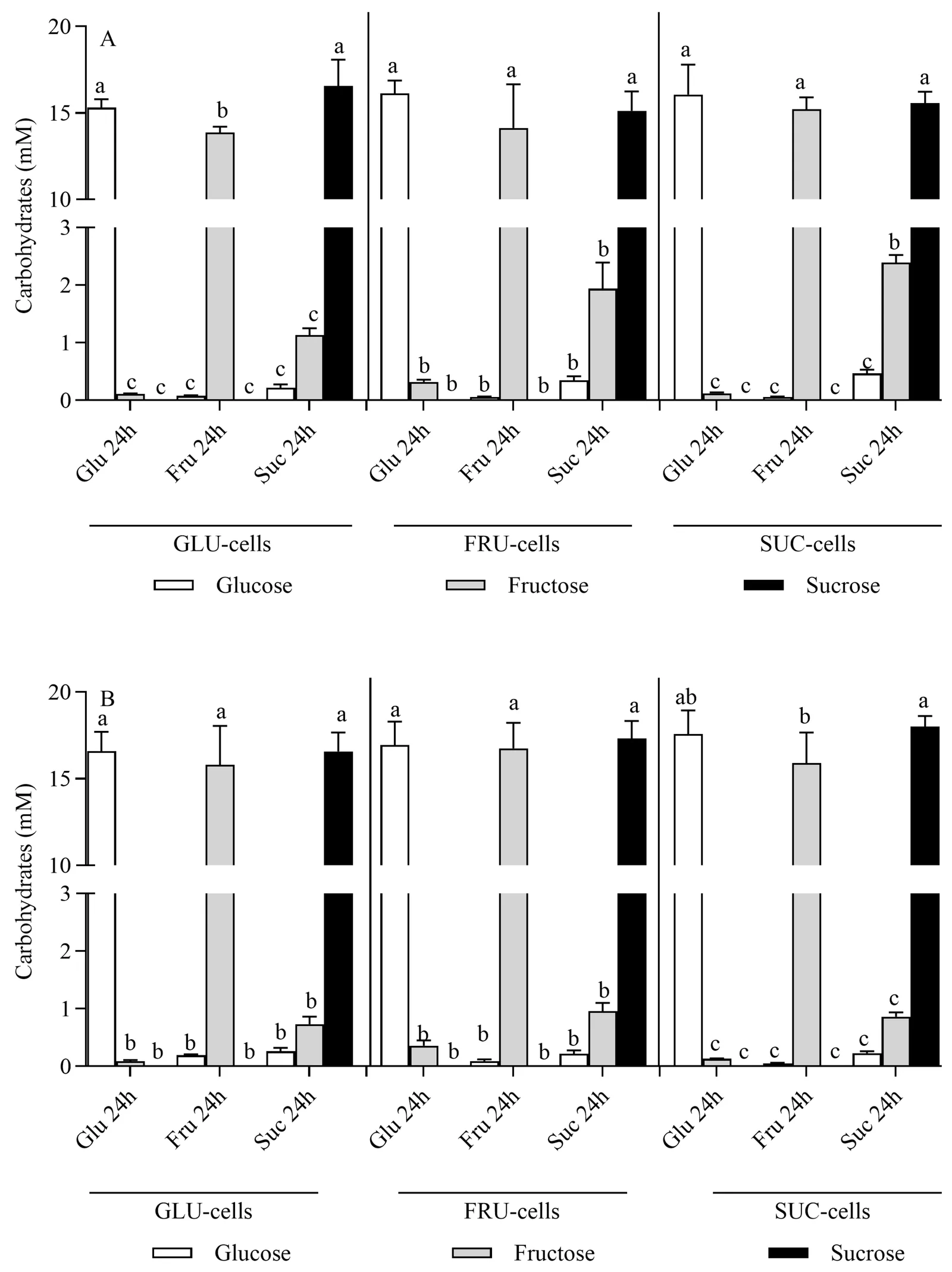
Sugar distribution following acute sugar exposure in differentiated GLU-, FRU-, and SUC-cells. Glucose, fructose, and sucrose concentrations measured after 24 h exposure to glucose, fructose, or sucrose in the (**A**) apical and (**B**) basolateral compartments. Data are expressed as mM and presented as means ± SD of four independently treated Transwell inserts obtained from two independent cell culture experiments (two inserts per condition in each experiment). Statistical analysis was performed by one-way ANOVA (all *p* < 0.001) followed by Tukey’s post hoc test. Different letters indicate statistically significant differences among sugars within each differentiated cell type.

To determine whether the phenotypic differences acquired during differentiation affected extracellular sugar distribution, glucose, fructose, and sucrose concentrations were measured in the apical and basolateral compartments after 24 h exposure to each sugar (Figure 8). Overall, the carbohydrate environment during differentiation did not markedly influence the extracellular sugar profiles observed after acute sugar exposure. In all cell types, the supplemented sugar was detected in comparable amounts in both the apical and basolateral compartments. Following sucrose exposure, appreciable amounts of glucose and, particularly, fructose were also detected in both compartments, consistent with sucrose hydrolysis.

Overall, these findings indicate that differentiation in the presence of a specific sugar did not result in a preferential extracellular distribution of that sugar or in detectable differences in sucrose hydrolysis under the experimental conditions used.

## 4. Discussion

The intestinal epithelium continuously adapts to changes in its nutritional environment, yet little attention has been paid to whether nutrient availability during epithelial differentiation contributes to programming the mature enterocyte functional characteristics. The present findings indicate that the carbohydrate composition of the differentiation environment is not merely a technical component required to support Caco-2 cell growth, but an active determinant of epithelial maturation. Although glucose, fructose, and sucrose all supported the development of functional epithelial monolayers, they generated distinct phenotypic and transcriptional profiles that persisted during a subsequent acute sugar challenge. Together, these findings identify the carbohydrate environment during differentiation as an underappreciated determinant of Caco-2 cell functional characteristics.

The first major finding of this study is that the carbohydrate environment experienced during differentiation influenced epithelial barrier maturation. Although Caco-2 cells differentiated successfully under all experimental conditions, sucrose promoted a more rapid acquisition of barrier integrity, as demonstrated by the earlier increase in TEER and the lower paracellular permeability to phenol red and FITC observed during differentiation. These differences progressively disappeared as differentiation progressed, indicating that all three culture conditions ultimately supported the formation of a functional epithelial monolayer, although with distinct maturation kinetics.

The effect of the carbohydrate environment on epithelial maturation was further reflected by the significantly lower dome density observed in SUC-cells at the end of differentiation. Dome formation is generally considered a hallmark of polarized epithelial cell function, resulting from vectorial ion and water transport across confluent monolayers, and depends on the balance between epithelial transport activity, cell–substrate adhesion, and paracellular permeability. Notably, previous studies have reported an inverse relationship between dome formation and transepithelial electrical resistance, whereby tighter epithelial barriers may reduce the accumulation of fluid beneath the cell monolayer [29]. The lower dome density observed in SUC-cells is therefore consistent with the enhanced barrier maturation detected during differentiation in this experimental group. However, dome formation is a multifactorial phenomenon and cannot be attributed solely to epithelial barrier properties. The accumulation of fluid beneath confluent epithelial monolayers may also depend on vectorial ion and solute transport, water movement, and the generation of local osmotic gradients. Indeed, osmotic gradients have been shown to drive epithelial dome formation and to involve aquaporin-mediated water transport [30]. Therefore, the markedly lower dome density observed in SUC-cells may reflect differences in one or more of these processes. Since the present study did not assess dome-fluid composition, aquaporin expression, or metabolic fluxes, the relative contribution of these mechanisms cannot be established and warrants further investigation. The asymmetric distribution of the sucrose hydrolysis products may also have contributed to this observation. As shown in Figure 5, glucose and fructose concentrations were higher in the apical than in the basolateral compartment of SUC-cells, consistent with the apical localization of sucrase-isomaltase. Complete hydrolysis of 25 mM sucrose into equimolar concentrations of glucose and fructose would theoretically increase the carbohydrate-related contribution to apical osmolality by up to approximately 25 mOsm compared with intact sucrose. This could generate a transient osmotic gradient influencing transepithelial water movement and, potentially, dome formation. However, total osmolality and water flux were not measured. Furthermore, at the same experimental time point, TEER and paracellular permeability to phenol red and FITC were comparable among GLU-, FRU-, and SUC-cells, providing no evidence of a persistent sucrose-induced closure of the paracellular pathway. Therefore, a causal relationship between asymmetric sucrose hydrolysis, osmotic water movement, and reduced dome formation cannot be established from the present data and should be investigated in specifically designed mechanistic studies.

The mechanisms underlying these effects remain to be elucidated. Increasing evidence indicates that epithelial differentiation and barrier function are influenced by nutrient-sensing pathways in addition to the structural organization of tight junctions. In particular, sweet taste receptors (T1R2/T1R3), which are expressed in the intestinal epithelium, have emerged as potential regulators of epithelial permeability and glucose transport. Artificial sweeteners have been shown to alter tight junction organization through T1R3-dependent mechanisms [31], while different dietary sugars display distinct affinities for sweet taste receptor subunits [32]. Although the present study did not investigate these signalling pathways, our findings suggest that the sugar composition of the differentiation medium may influence epithelial maturation through mechanisms that extend beyond its role as a source of metabolic substrates.

A second major finding of this study is that the sugar environment during differentiation markedly influenced the expression of genes involved in sugar digestion and transport. Rather than inducing the expression of a single transporter, glucose, fructose, and sucrose generated distinct transcriptional profiles, suggesting that the carbohydrate environment contributes to shaping the differentiated characteristics of intestinal epithelial cells.

The expression of intestinal sugar transporters is highly dynamic and depends on both luminal nutrient availability and intracellular metabolic signalling. Previous studies have shown that fructose and sucrose modulate the expression of glucose transporters and digestive enzymes in intestinal cell models, although the reported responses have not always been consistent. For example, Le Gall et al. reported an increased expression of SLC2A2 (encoding GLUT2) mRNA levels following fructose exposure and enhanced SI promoter activity after fructose or sucrose treatment in differentiated Caco-2/TC7 cells [13], whereas Gouyon et al. [14] reported fructose-induced stabilization of SLC2A5 (encoding GLUT5) mRNA in differentiated Caco-2 cells. These apparent discrepancies most likely reflect differences in experimental design, as previous studies primarily investigated short-term responses in already differentiated intestinal cells, whereas the present study examined the influence of the carbohydrate environment throughout the entire differentiation process.

One of the principal regulators of this adaptive response is the ChREBP, a transcription factor that coordinates the expression of genes involved in carbohydrate metabolism in several metabolic tissues, including the intestine. Both glucose and fructose promote ChREBP activation and nuclear translocation, leading to increased expression of genes involved in glycolysis, fructolysis, lipogenesis, and intestinal sugar transport [15]. Moreover, intestine-specific deletion of ChREBP in mice markedly reduces the expression of GLUT2, GLUT5, and SGLT1, supporting its role as a central regulator of intestinal carbohydrate handling [33,34]. Although ChREBP activation was not investigated in the present study, the distinct transcriptional profiles observed in GLU-, FRU-, and SUC-cells are consistent with the existence of a nutrient-dependent transcriptional adaptation occurring during epithelial differentiation.

Besides intracellular nutrient sensing, extracellular sugar sensing may also contribute to these adaptive responses. Sweet taste receptors expressed by intestinal epithelial cells have been implicated in the regulation of SGLT1 and GLUT2 expression in response to luminal sugars [35]. Whether these receptors participate in the long-term modulation of intestinal differentiation remains unknown, but they represent another plausible mechanism through which the carbohydrate environment during differentiation could influence the functional characteristics of differentiated Caco-2 cells.

The phenotypic differences established during differentiation were reflected in distinct responses to acute sugar exposure, although not all cellular functions were affected to the same extent. Acute exposure to glucose, fructose, or sucrose did not affect barrier integrity or cell viability, indicating that the differentiated phenotype remained stable throughout the 24-h challenge. In contrast, the transcriptional response of sugar-related genes and, to a lesser extent, dome formation were influenced by both the differentiation medium and the sugar used for the challenge, demonstrating that the nutritional history of the cells modulates their adaptive response to changes in sugar availability.

Despite the distinct transcriptional responses, no marked differences in extracellular sugar distribution were detected after acute exposure. Indeed, NMR analysis showed comparable sugar distribution between the apical and basolateral compartments irrespective of the sugar used during differentiation. Likewise, differentiation in glucose, fructose, or sucrose did not modify the apparent capacity of Caco-2 cells to hydrolyse sucrose, as indicated by the comparable appearance of glucose and fructose following sucrose exposure. Together, these findings suggest that the transcriptional adaptations induced during differentiation are not necessarily translated into detectable differences in extracellular sugar distribution under the experimental conditions adopted in this study.

This apparent discrepancy between gene expression and sugar transport is not unexpected. Changes in mRNA abundance do not necessarily reflect transporter activity, membrane localization, or transport capacity, all of which are regulated by multiple post-transcriptional and post-translational mechanisms. However, the absence of detectable differences in extracellular sugar distribution does not exclude differences in intracellular sugar handling or transporter activity. Although ^1^H NMR spectroscopy would allow a broader characterization of the intracellular and extracellular metabolome, the present analysis was intentionally focused on the extracellular concentrations of glucose, fructose, and sucrose. Further studies will be required to clarify the functional consequences of the transcriptional adaptations observed here.

The present study has some limitations. The analysis of sugar transporters and sucrase-isomaltase was limited to transcript levels; protein abundance, membrane localization, and transporter activity were not directly assessed. Therefore, the functional consequences of the observed transcriptional adaptations require confirmation at the protein level and through direct assessment of transporter localization and activity in future studies. In addition, the molecular mechanisms underlying the observed phenotypic differences, including ChREBP and sweet taste receptor signalling, were not directly investigated. It should also be acknowledged that, although the three sugars were supplied at the same molar concentration, sucrose potentially provides twice the number of monosaccharide units following complete hydrolysis. Therefore, differences in monosaccharide availability after sucrose hydrolysis may have contributed to some of the effects observed in SUC-cells and cannot be completely disentangled from those related to carbohydrate identity. Nevertheless, the integration of barrier function, dome formation, gene expression, and ^1^H-NMR-based analysis of extracellular sugar distribution provided a comprehensive functional, molecular, and metabolic characterization of how the carbohydrate environment during differentiation influences the functional characteristics of intestinal epithelial cells.

Collectively, these findings demonstrate that the carbohydrate environment experienced during epithelial differentiation contributes to shaping the functional characteristics of intestinal epithelial cells. From a methodological perspective, they identify the carbohydrate composition of the differentiation medium as a biological variable that should be carefully controlled and reported to improve the design, reproducibility, and comparability of studies using differentiated Caco-2 cells. More broadly, although obtained in an established in vitro model, these findings support the concept that nutrient availability during epithelial maturation may contribute to intestinal adaptation to changes in dietary carbohydrate exposure and provide a mechanistic framework for future studies addressing this hypothesis in vivo.

## Data Availability

The raw data supporting the conclusions of this article will be made available by the authors on request.

## Abbreviations

The following abbreviations are used in this manuscript:

ANOVA	Analysis of variance
ChREBP	Carbohydrate-responsive element-binding protein
DMEM	Dulbecco’s Modified Eagle Medium
DPBS	Dulbecco’s phosphate-buffered saline
DSS	2,2-dimethyl-2-silapentane-d6-5-sulfonate
FITC	Fluorescein isothiocyanate
FRU	Fructose
GLU	Glucose
LDH	Lactate dehydrogenase
MTT	Methylthiazolyldiphenyltetrazolium bromide
NADH	Nicotinamide adenine dinucleotide
NMR	Nuclear magnetic resonance
PR	Phenol red
SD	Standard deviation
SI	Sucrase-isomaltase
SLC2A1	Solute carrier family 2 member 1
SLC2A2	solute carrier family 2 member 2
SLC2A5	solute carrier family 2 member 5
SLC5A1	solute carrier family 5 member 1
SUC	Sucrose
TEER	Transepithelial electrical resistance
